# Anti-inflammatory effects and molecular mechanisms of bioactive small molecule garlic polysaccharide

**DOI:** 10.3389/fnut.2022.1092873

**Published:** 2023-01-09

**Authors:** Xin Shao, Jialong Li, Huidan Zhang, Xuhui Zhang, Chongzhen Sun, Xin Ouyang, Yong Wang, Xiyang Wu, Chunbo Chen

**Affiliations:** ^1^Department of Critical Care Medicine, Maoming People's Hospital, Maoming, China; ^2^Department of Food Science and Engineering, Jinan University, Guangzhou, China; ^3^Department of Intensive Care Unit of Cardiac Surgery, Guangdong Cardiovascular Institute, Guangdong Provincial People's Hospital, Guangdong Academy of Medical Sciences, Guangzhou, China; ^4^Department of Critical Care Medicine, Shenzhen People's Hospital, Shenzhen, China

**Keywords:** small molecule garlic polysaccharide, anti-inflammatory, molecular mechanisms, bioactive, NF-κB, STAT3

## Abstract

Although garlic polysaccharides have been found to possess anti-inflammatory activities, anti-inflammatory study on small molecule water-soluble garlic polysaccharide (WSGP) is few. In this study, a novel WSGP with a molecular weight of 1853 Da was isolated by DEAE-52 and Sephadex G-100 column and the chemical composition was identified by monosaccharide composition and methylation analysis. Furthermore, the antioxidant effects of WSGP and the potential molecular mechanisms on LPS-induced inflammatory responses in RAW264.7 macrophage cells were investigated. The results showed that WSGP has strong antioxidant activity, such as DPPH, hydroxyl, superoxide anion, ABTS radical scavenging capacity, Fe^2+^ chelating ability and reducing power. Meanwhile, WSGP could considerably suppress the manufacturing of NO and the mRNA and protein expression degrees of IL-6, TNF-α, and IL-1β in LPS inspired RAW264.7 macrophages WSGP could significantly suppress the production of NO and the mRNA and protein expression levels of IL-1β, IL-6, and TNF-α in LPS stimulated RAW264.7 macrophage cells (*p* < 0.05). In addition, the phosphorylated IκB-α, p65, and STAT3 proteins were significantly increased in LPS-induced macrophages, while this trend was significantly reversed by WSGP treatment in a concentration-dependent manner (*p* < 0.05). Consequently, WSGP supplementation might reduce LPS-induced inflammatory responses by suppressing proinflammatory cytokines and NF-κB and STAT3 pathway activation. The finding of this research would give scientific guidelines for the judicious use of small molecular garlic polysaccharide in anti-inflammatory treatments.

**Graphical Abstract:**
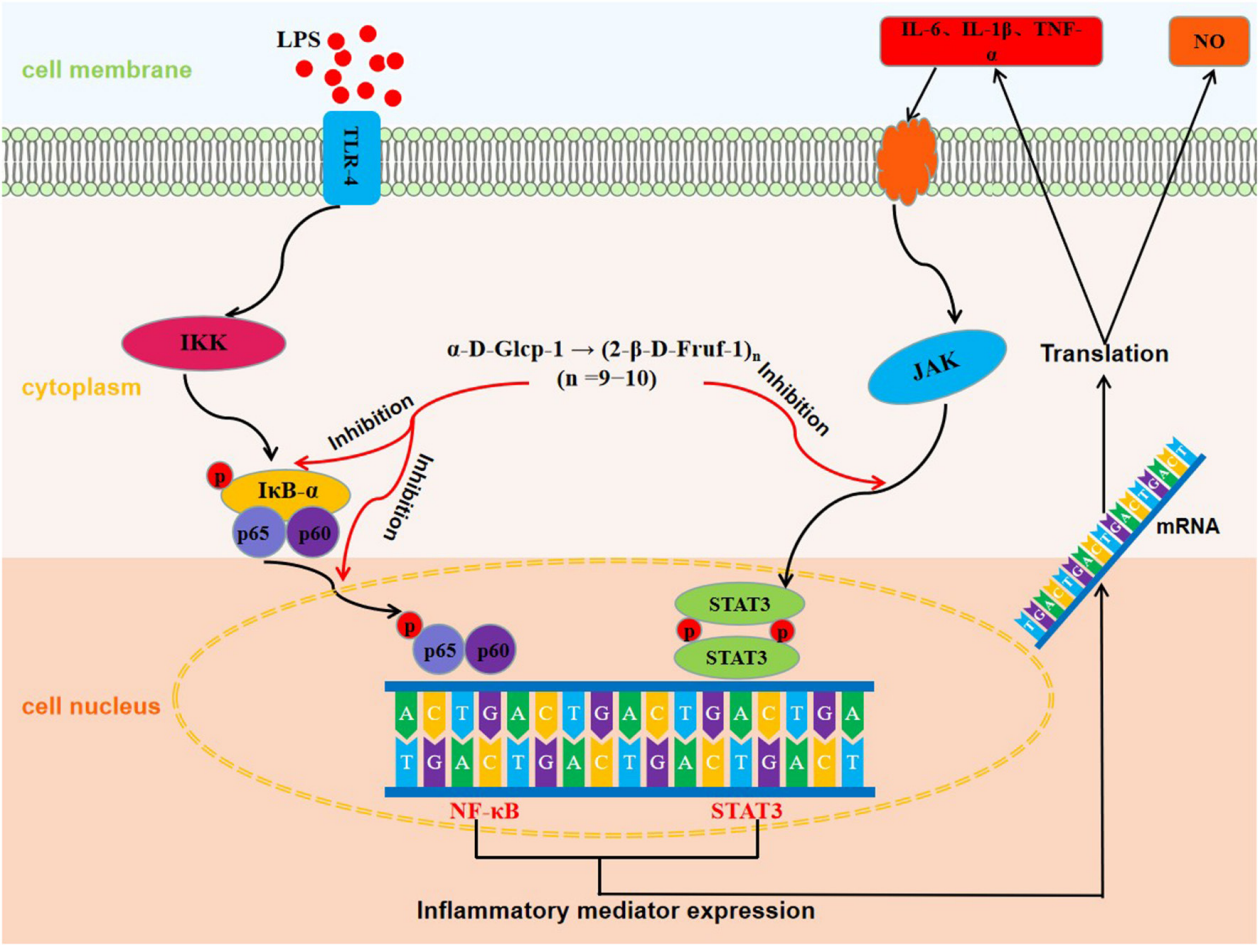
The molecular mechanisms of anti-inflammatory effect of WSGP.

## 1. Introduction

As a member of the Liliaceae family, garlic is both a functional and edible plant (1). It has been used as a spice, vegetable, and in traditional medicine for centuries owing to its distinctive flavor and health advantages (2). Nowadays, garlic has attracted increasing attention from scientists, mainly owing to its varying biological activities, including antioxidant, immune-boosting, anti-virus, and anti-bacterial properties (3). Garlic is rich in bioactive compounds, such as polysaccharides and flavonoids, among which polysaccharides are one of the major active constituents, accounting for approximately 80% of the dry weight (4). Notably, the antioxidant and anti-inflammatory activities are the significant properties of garlic polysaccharides (5, 6). It has been observed that the antioxidant mechanism of garlic polysaccharides *in vitro* involves two components: binding to the metal ions to minimize the generation of free radicals and interacting with distinct free radicals directly to produce the scavenging action. Recently, clinical studies have shown that garlic polysaccharide alleviates the development of colitis by inhibiting proinflammatory cytokines and regulating gut microbiota (7). However, these studies were about garlic polysaccharides with molecular weight over 10 kDa.

A proper inflammatory response protects living tissues from damaging stimuli and revitalizes the immune system-mediated healing process, and inflammation is a crucial and complicated physiological defense process for the body to withstand external stimuli (8, 9). However, chronic inflammatory illnesses such inflammatory bowel disease, heart disease, diabetes, and cancer may be aided by an overabundance of pro-inflammatory cytokines (10, 11). Macrophages are effector cells of the innate immune system that play an important role in maintaining homeostasis and regulating inflammatory responses, tissue remodeling, and repair (12, 13). In the inflammatory response, macrophages play a significant role in pathogen resistance and inflammation resolution by secreting a large array of inflammatory mediators (14, 15).

For *in vitro* research, the RAW264.7 murine macrophage cell line is considered to be the most effective anti-inflammatory cell type (16). Lipopolysaccharide (LPS) can be recognized by receptors of macrophages, thus inducing inflammatory responses in macrophages (17, 18). As an indispensable receptor for LPS identification and binding, toll-like receptor-4 (TLR-4) can induce simultaneous activation of various signaling pathways including nuclear factor-kappa B (NF-κB) and signal transducer and activator of transcription3 (STAT3) (19). This advances noteworthy synthesis of pro-inflammatory factors including nitric oxide (NO), interleukin-6 (IL-6), interleukin-1β (IL-1β), tumor rot factor-α (TNF-α), which initiate a cascade of reactions and aggravate the inflammatory response (20). As a result, it is assumed that inhibiting pro-inflammatory cytokine and mediator production is a critical strategy in the prevention of inflammation (21). Currently, antibiotic drugs such as Aerosporin, dexamethasone, and prednisone are used in therapies for inflammatory responses. However, the long-term use of these drugs has revealed a narrow therapeutic index and considerable adverse effects, such as fever and hepatorenal syndrome, severely restricting their application in clinical practice (22, 23). Therefore, using natural bioactivties from food resources with outstanding anti-inflammatory effects and low toxic side effects as supplements would be a good alternative.

As a natural plant polysaccharide, garlic polysaccharides have found to possess anti-inflammatory activity. However, studies on the anti-inflammatory signaling pathway mechanism of garlic polysaccharide *in vitro* and the inflammatory property of the garlic polysaccharide with small molecular weight toward macrophages have not been conducted yet. Previously, we extracted a novel small molecule water-soluble garlic polysaccharide (WSGP) with a molecular weight of 1853 Da and analyzed its structure (6). Meanwhile, our results showed that WSGP could relief the colitis symptoms of dextran sodium sulfate (DSS) induced mice. In this work, the antioxidant and anti-inflammatory properties along with the underlying anti-inflammatory mechanisms of WSGP were elucidated in greater detail. Herein, the antioxidant activity of WSGP was evaluated regarding DPPH, hydroxyl, superoxide anion, ABTS radical scavenging capacity, Fe^2+^ chelating ability and reducing power. The potential inhibitory effect of WSGP on LPS-induced RAW264.7 was also investigated. Furthermore, in order to confirm the inhibitory effect of WSGP on the NF-κB/STAT3 signaling pathway in the inflammatory response of RAW264.7 macrophages, the phosphorylation levels of IκB-α, p65, and STAT3 proteins were measured by Western Blotting assay.

## 2. Materials and methods

### 2.1. Materials and cell culture

Jinxiang garlic was purchased from Jinxiang County Garlic Research Institute (Shandong, China). ELISA kits of IL-6, TNF-α, and IL-1β were bought from Neobioscience Technology Co. Ltd. (Shenzhen, China). 3-(4,5-dimethylthiazol-2-yl)-2,5-diphenyltetrazolium bromide (MTT), dimethyl sulfoxide (DMSO), LPS, phosphate-buffered saline (PBS), and Dulbecco's modified eagle's medium (DMEM) were acquired from Aladdin Co. (Shanghai, China). The RAW264.7 macrophage cell line was obtained from the Cell Bank of the Shanghai Institute of Cell Biology and Biochemistry, Chinese Academy of Sciences (Shanghai, China). Specific primary antibodies against p-IκBα (#3033), IκBα (#4812), p-p65 (#3031), p65(#8242), p-STAT3(#9145), STAT3(#12640), and GAPDH (#5174) were purchased from Abcam (Cambridge, UK). The cells were maintained in DMEM containing 100 U/mL penicillin, 100 μg/mL streptomycin, 2 mmol/L glutamine, and 10% fetal bovine serum (FBS). Cells were cultured at a 37°C humidified atmosphere of 5% CO_2_. RAW264.7 macrophages were cultured in DMEM supplement with 10% fetal bovine serum, 100 U/mL penicillin, 100 g/mL streptomycin, and 2 mmol/L glutamine (FBS). Cells were grown in a 37°C, humidified, 5% CO_2_ environment.

### 2.2. Extraction and isolation of WSGP

The fresh garlic was obtained from the Jinxiang Garlic Research Institute, and the WSGP was prepared according to our previously reported method (6). Briefly, after early extraction and rotary evaporation concentration, the precipitate was collected, diluted in water again, and then subjected to a series of progressive precipitations using ethanol at 70, 80, and 90% concentrations. Thereafter, the crude water-soluble garlic polysaccharide (CWSGP) was obtained by removing protein by the Sevag method and the extract was separated and purified by using cellulose column (DEAE-52, 5.5 × 30 cm) and Sephadex G-100 column (1.6 × 60 cm) to afford WSGP.

### 2.3. Structural analysis of WSGP

In accordance with our previous, we analyzed the monosaccharide content and methylation status of WSGP (6). After the above procedures, the structural formula of the small garlic polysaccharide was finally determined.

### 2.4. Determination of *in vitro* antioxidant activity of WSGP

WSGP solutions with different concentrations (0.125–4.0 mg/mL) were prepared with distilled water for the following *in vitro* antioxidant assays. As positive and blank controls, respectively, Vc and distilled water were utilized. The OD value was determined with the use of a UV-visible spectrophotometer (Evolution 220; Thermo Fisher, CA, USA).

#### 2.4.1. Determination of scavenging ability of WSGP on DPPH free radical

The experimental procedure is slightly modified based on Chen et al.'s method (24). In the dark, 3.0 mL of WSGP solution and 1.0 mL of 0.4 mmol/L DPPH anhydrous ethanol solution were combined for 20 min at room temperature. The DPPH radical scavenging effectiveness of WSGP was determined using Formula (1):


(1) $$\begin{array}{l} \begin{array}{c} Y_{1}\left(\%\right)=\left(1-\frac{A-A_{0}}{A_{1}}\right)\times 100 \end{array} \end{array}$$

Where *Y*_1_ is the DPPH free radical scavenging ability (%); where *A* is the OD of WSGP sample; *A*_0_ is the OD of deionized water replacing ethanol solution; *A*_1_ is the OD of deionized water replacing WSGP sample.

#### 2.4.2. Determination of hydroxyl radical scavenging ability of WGSP

The experimental procedure is slightly modified based on previous method (25). For 50 min at 40 °C, we combined 1.0 mL of WSGP solution, 1.0 mL of 0.75 mmol/L phenanthroline, 1.0 mL of 0.75 mmol/L FeSO4, 1.0 mL of 0.01% (V/V) H2O2, and 1.5 mL of 0.15 mol/L sodium phosphate, pH 7.4. The OD value was obtained at 536 nm. Formula (2) was used to determine the scavenging effectiveness of WSGP on the hydroxyl radical.


(2) $$\begin{array}{l} \begin{array}{c} Y_{2}\left(\%\right)=\left(\frac{A-A_{1}}{A_{0}-A_{1}}\right)\times 100 \end{array} \end{array}$$

Where *Y*_2_ is the hydroxyl radical scavenging ability (%); where *A* is the OD of WSGP sample; *A*_0_ is the OD of deionized water replacing H_2_O_2_ solution; *A*_1_ is the OD of deionized water replacing WSGP sample.

#### 2.4.3. Determination of reducing the ability of WSGP

The experimental procedure is slightly modified based on Chen et al.'s method (26). 1.0 mL of WSGP solutions, 2.5 mL of 0.20 mol/L sodium phosphate with pH 6.6, and 1.0 mL of 1% (W/V) potassium ferricyanide were mixed in a 40°C incubator for 40 min. After being cooled to room temperature, before centrifuging at 5,000 rpm for 5 min, 2.5 mL of 10% (W/V) trichloroacetic acid was added and then 2.5 mL of supernatant was taken, and 0.5 mL of 1% (W/V) ferric chloride and 2.5 mL of distilled water were added. The OD value was estimated at 700 nm. The scavenging efficiency of garlic polysaccharide on hydroxyl radical was calculated according to Formula (3):


(3) $$\begin{array}{l} \begin{array}{c} Y_{3}=A-A_{0} \end{array} \end{array}$$

Where *Y*_3_ is the reducing ability; where *A* is the OD of WSGP sample; *A*_0_ is the OD of deionized water replacing ferric chloride solution.

#### 2.4.4. Evaluation of WSGP's capacity to scavenge ABTS free radical

The experimental procedure is slightly modified based on Erel's method (27). 1.0 mL of WSGP solution and 4.0 mL of ABTS+· solution was mixed in a centrifuge tube. The OD value was calculated at 734 nm. We used the formula (1) to determine WSGP's ability to scavenge ABTS free radical. *A* is the OD of WSGP sample, *A*_0_ is the OD of deionized water replacing ABTS+· solution, *A*_1_ is the OD of deionized water replacing WSGP sample.

#### 2.4.5. The ability of WSGP to scavenge superoxide anion radical

The experimental procedure is slightly modified based on Bi et al.'s method (28). In the dark, 50 μL of the solution, 50 μL of 300 μmol/L 4-Nitro blue tetrazolium chloride (NBT) solution, 50 μL of 936 μmol/L NADH and 50 μL of 120 μmol/L PMS were added into 96-well plates to incubate at room temperature for 5 min. The OD value was determined at 569 nm. The superoxide anion radical scavenging rate of WSGP was measured by the formula (1), where *A* is the OD of WSGP sample, *A*_0_ is the OD of deionized water replacing NBT solution, and A1 is the OD of deionized water replacing WSGP sample.

#### 2.4.6. Determination of Ferric ion-chelating ability of WSGP

The experimental procedure is slightly modified based on Dinis et al.'s method (29). We mixed 2.0 mL of WSGP solutions, 0.1 mL of 2 mmol/L FeCl2 and 3.7 mL of distilled water in a centrifuge tube for 5 min. Afterward, 0.2 mL of 5 mmol/L ferrozines was added. After incubating the mixture for 10 min at 25°C, the absorbances were measured at 562 nm. The mixture was incubated for 10 min at 25°C before measuring their absorbances at 562 nm. The Fe^2+^ chelating capacity of polysaccharides was also calculated according to the formula (1), where A is the OD of WSGP sample, *A*_0_ is the OD of deionized water replacing ferrous chloride solution, *A*_1_ is the OD of deionized water replacing WSGP sample.

### 2.5. Effects of WSGP on LPS-induced RAW264.7 macrophages

#### 2.5.1. Determination of cell viability

The MTT assay was utilized to determine the RAW264.7 macrophages capacity to proliferate. Briefly, RAW264.7 macrophages were cultured on a 96-well plate (5 × 104 cells/well) at 37 °C humidified atmosphere of 5% CO_2_ for 24 h. After 24 h of treatment with WSGP (0, 62.5, 125, 250, 500, and 1,000 μg/mL), RAW264.7 macrophages were exposed to 10 μL of MTT solution (5 mg/mL) for 4 h. After that, 150 μL of DMSO was used to dissolve the crystal. Cell viability rates were calculated based on the absorbance at 570 nm.

#### 2.5.2. Griess test

The Griess test was used to quantify the amount of nitrite in the growth media, as this is the mechanism by which nitric oxide (NO) is generated by the cells. The cells were incubated in 96-well plates (5 × 104 cells/well) and cultured overnight with 5% CO_2_, then pretreated with WSGP (0, 62.5, 125, 250, 500, and 1,000 μg/mL) for 3 h before treatment with 1 μg/mL LPS for 24 h. The NO levels were measured by collecting the cell supernatants after 10 min of incubation and using a NO assay kit (Sigma-Aldrich, CA, USA). The OD value was detected at 540 nm.

#### 2.5.3. Enzyme-linked immunosorbent assay

Following a 24 h incubation period in 6-well plates at a density of 4 × 105 cells/well, RAW264.7 macrophages were pretreated with WSGP (0,62.5,125,250,500, and 1,000 g/mL) for 3 h before being exposed to 1 μg/mL LPS for another 24 h. The RAW264.7 macrophages were plated in 6-well plates (4 × 105 cells/well) and incubated for 24 h, then pretreated with WSGP (0, 62.5, 125, 250, 500, and 1,000 μg/mL) for 3 h before treatment with 1 μg/mL LPS for 24 h. After incubation, cells were centrifugated at 3,000 rpm at 4°C for 20 min. Cell supernatants were tested using an ELISA kit (Xinbosheng, Shenzhen, China) to detect the levels of cytokines (IL-1β, IL-6, and TNF-α) secreted by the cells.

#### 2.5.4. RT-PCR analysis

The RAW264.7 macrophages with 4 × 105 cells/well were inoculated into 6-well plates and pretreated with WSGP (0, 62.5, 125, 250, 500, and 1,000 μg/mL) for 3 h before treatment with 1 μg/mL LPS for 24 h. Total RNA was isolated with TrizolTM reagent. cDNA was synthesized using a PrimeScript™ RT Reagent Kit with gDNA Eraser Reverse Transcription Kit (Takara, Tokyo, Japan). RT-PCR with TB Green^®^ Premix DimerEraserTM (Takara, Tokyo, Japan) was used to get ready the mRNA expression. The primer sets used were as follows: One cycle of denaturation was carried out at 95°C for 30 s, and then the denaturation continued at 95°C for 5 s. The initial temperature was annealed to 55°C for 30 s and then heated to 72°C for 30 s. There were 40 cycles in this process. The ABI PRISM^®^ 7,300 Sequence Detection System (Applied Biosystems, Canada) was used to conduct the reactions. The outcomes were represented as RQ = 2–ΔΔCt. The primer sequences are provided in Table 1.

**Table 1:** Sequences of primers used for RT-PCR.

**Gene**	**Forward primer (5'-3')**	**Reverse primer (5'-3')**
GAPDH	CCTCGTCCCGTAGACAAAATG	TCTCCACTTTGCCACTGCAA
IL-1β	AGCTCTCCACCTCAATGGAC	GACAGGCTTGTGCTCTGCTT
IL-6	TCGTGGAAATGAGAAAAGAGTTG	AGTGCATCATCGTTGTTCATACA
TNF-α	CTACTGAACTTCGGGGTGAT	CAGGCTTGTCACTCGAATT

#### 2.5.5. Western blotting assay

The RAW264.7 macrophages were plated in 6-well plates (4 × 10^5^ cells/well) and incubated for 24 h, then pretreated with WSGP (0, 62.5, 125, 250, 500, and 1,000 μg/mL) for 3 h before treatment with 1 μg/mL LPS for 24 h. The cells were collected after incubation and then lysed in RIPA cell lysis solution for 1 h while kept cold in the freezer. The BCA Protein Assay Kit (Sigma-Aldrich^®^, MO, USA) was used to quantify the protein concentrations. Proteins of equivalent amounts were separated on 10% SDS-PAGE and transferred to PVDF membranes for analysis. The membranes were blocked with 5% non-fat milk and then treated with primary antibodies (p-IκBα, IκBα, p-p65, p65, p-STAT3, STAT3, and GAPDH) at 4°C overnight. PVDF membranes were treated with appropriate secondary antibodies (Abcam, Cambridge, UK) for 1 h after three washes with TBS solution. The blots were washed with TBS solution three times. Signals were detected using Enhanced Chemiluminescence (ECL) detection (Thermo, MA, USA). Image J software (Bethesda, Maryland, USA) was used to quantify the blot density. TBS solution was used to wash the blots three times. Enhanced chemiluminescence (ECL) detection was used to pick up the signals (Thermo, MA, USA). The blot density was quantified using Image J software (Bethesda, Maryland, USA).

### 2.6. Statistical analysis

The data is shown as the mean ± SD. Except when noted otherwise, all assays were conducted at least three times. One-way analysis of variance (ANOVA) was carried out. Utilizing GraphPad Prism version 8.0 (GraphPad Software, Inc. San Diego, CA, USA), Pearson's correlation was calculated.

## 3. Results and discussion

### 3.1. Chemical composition of WSGP

**Figure 1:**
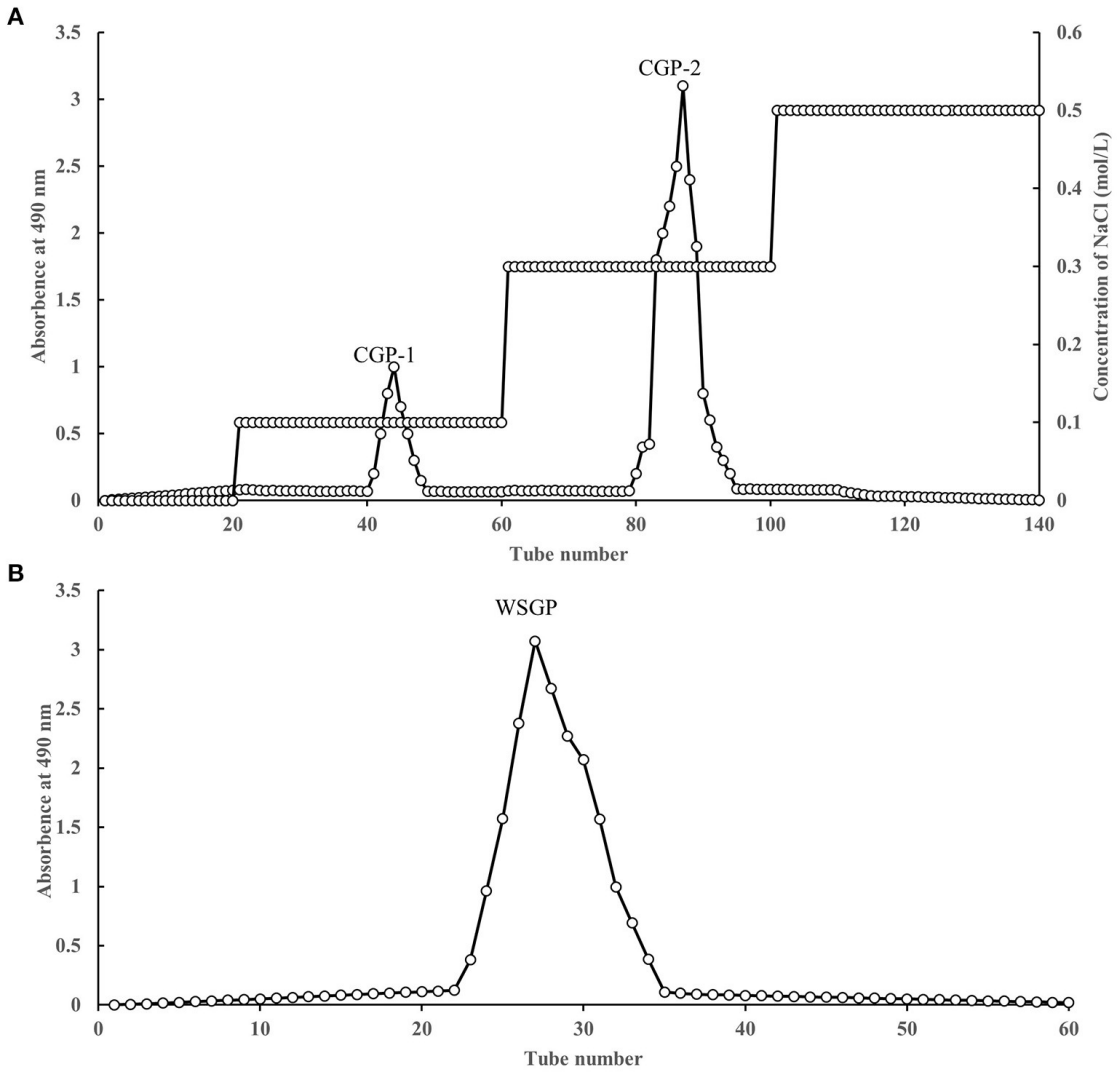
Stepwise elution curve of crude polysaccharides on DEAE-52 column **(A)** and elution curve of polysaccharides fraction (F-1) on SephadexG-100 column **(B)**.

Two fractions were accomplished by adding the CWSGP to a DEAE-52 cellulose column. The CWSGP was loaded onto a DEAE-52 cellulose column to obtain two fractions. As Figure 1A shows, two eluting peaks were obtained by gradient elution of garlic polysaccharide CWSGP with different concentrations of NaCl solution (0.1 and 0.3 mol/L NaCl), and they were named CGP-1 and CGP-2 respectively. Based on the CWSGP loading quantity, the yield of CGP-1 and CGP-2 were 4.23 and 23.46%, respectively. CGP-2 was primarily chosen for further purification using a Sephadex G-100 gel filtration column to get a single peak designated WSGP due to the poor yield of CGP-1 (Figure 1B). The yield of WSGP was 83.51% based on the CGP-2 loading quantity. High-performance anion exchange chromatography (HPAEC) was utilized to determine the monosaccharide content of WSGP.

**Figure 2:**
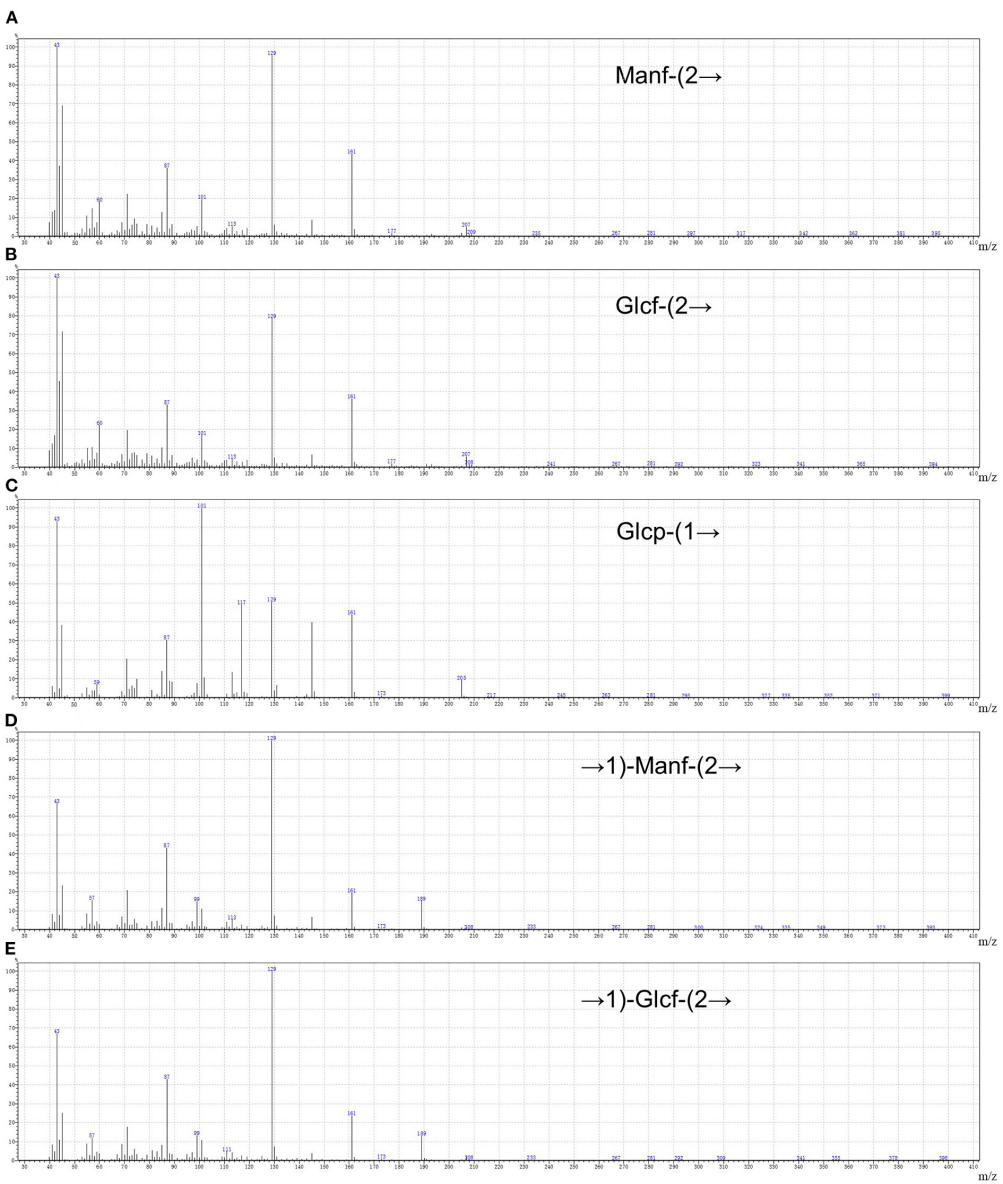
The five methylated sugar residues of WSGP by Using GC–MS. 1,3,4,5-Me_4_-Manf **(A)**, 1,3,4,5-Me_4_-Glcf **(B)**, 2,3,4,6-Me_4_-Glcp **(C)**, 3,4,5-Me_3_-Manf **(D)**, and 3,4,5-Me_3_-Glcf **(E)**. The data were expressed as means ± SD from three independent experiments.

By drawing the standard curve of glucan standard product, we determined that the relative molecular weight of WSGP was 1853Da, which was several times or even tens of times different from the relative molecular weight of garlic polysaccharide in previous studies (4, 5). Table 2 demonstrates that WSGP is a heteropolysaccharide with fructose (64.90%) as the predominate monosaccharide component. WSGP also included small amounts of fucose (2.60%) and glucose (32.50%). Methylation combined with Gas Chromatography-Mass Spectrometer (GC-MS) examination might determine the linkage pattern of the monosaccharide residues. As shown in Figure 2 and Table 3, five types of partly methylated alditol acetates were identified, 1,3,4,5-Me_4_-Manf, 1,3,4,5-Me_4_-Glcf, 2,3,4,6-Me_4_-Glcp, 3,4,5-Me_3_-Manf and 3,4,5-Me_3_-Glcf, which were assigned to Manf-(2 → (1.20 %), Glcf-(2 → (1.11 %), Glcp-(1 → (15.13 %), → 1)-Manf-(2 → (40.26 %) and → 1)-Glcf-(2 → (42.30 %) residues, respectively. Since fructose is a ketose that isomerizes into mannose and glucose during reduction and then into mannose and glucoside of the furan ring, mannose residues are detected in WSGP methylation study. Combining monosaccharide composition analysis and methylation analysis as well as the ^13^C NMR and ^1^H NMR and two-dimensional NMR results of WSGP reported previously, we can infer that the structural formula of WSGP is α-D-Glcp-1 → (2-β-D-Fruf-1) n → (n =9–10) (6).

**Table 2:** Monosaccharide composition of WSGP.

**Monosaccharide**	**RT**	**Standard curve**	**R^2^**	**Molar ratio (%)**
Fucose	5.825	y = 0.183x + 1.03	0.994	2.60
Glucose	17.817	y = 0.267x + 1.493	0.996	32.50
Fructose	24.959	y = 0.106 + 0.276	0.996	64.90

**Table 3:** Methylation analysis of the WSGP.

**Methylated sugar**	**Mass fragments (m/z)**	**Molar ratios (%)**	**Type of linkage**
1,3,4,5-Me_4_-Manf	87,101,129,145,161	1.20	Manf-(2 →
1,3,4,5-Me_4_-Glcf	87,101,129,145,161	1.11	Glcf-(2 →
2,3,4,6-Me_4_-Glcp	43,71,87,101,117,129,145,161,205	15.13	Glcp-(1 →
3,4,5-Me_3_-Manf	43,71,87,99,101,129,145,161,189	40.26	→ 1)-Manf-(2 →
3,4,5-Me_3_-Glcf	43,71,87,99,101,129,145,161,189	42.30	→ 1)-Glcf-(2 →

### 3.2. The *in vitro* antioxidant activity of WSGP

**Figure 3:**
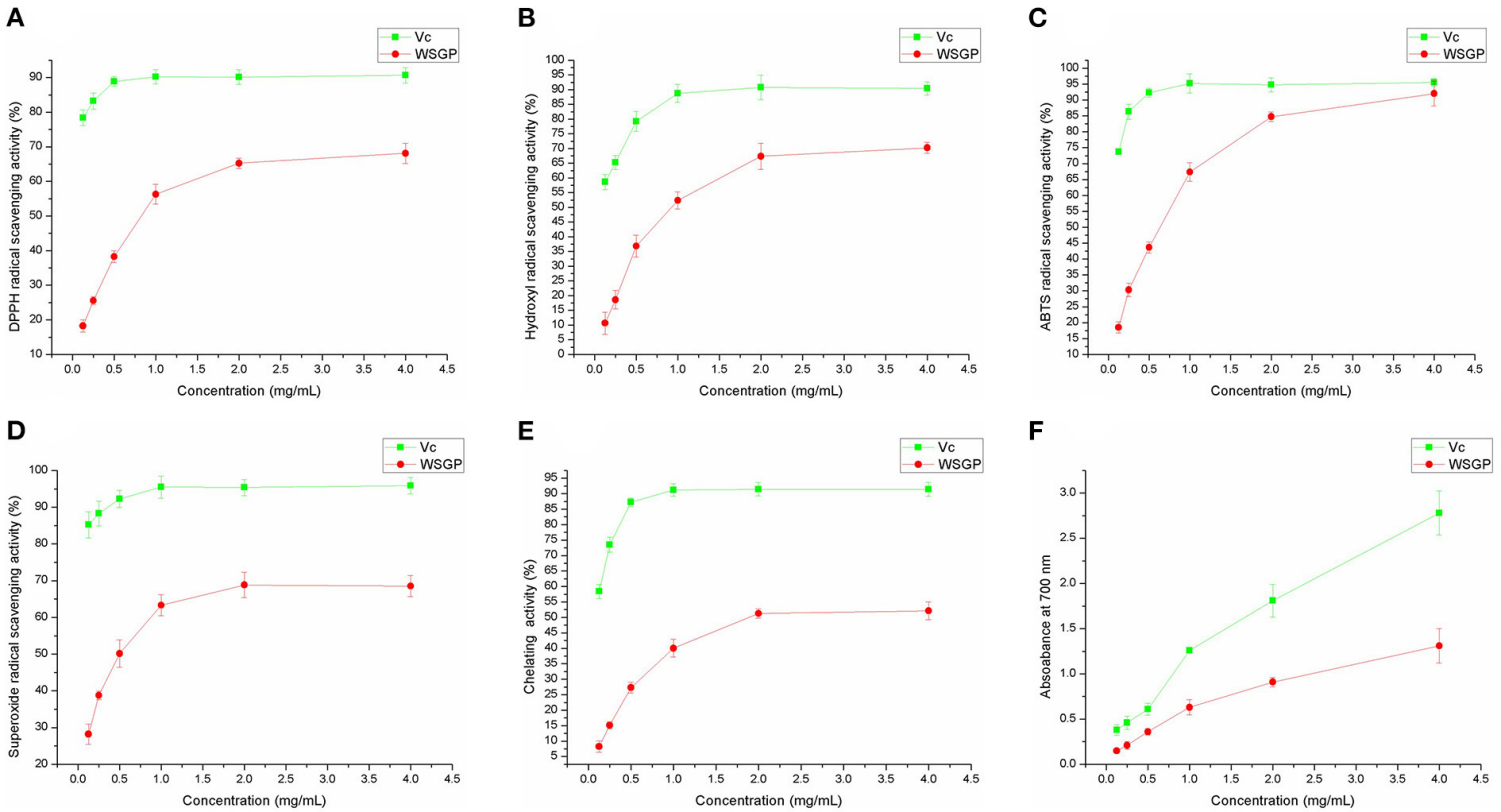
The antioxidant abilities of WSGP include DPPH **(A)**, ABTS **(B)**, hydroxyl **(C)**, and superoxide **(D)** radical scavenging powers, chelating ability on Fe^2+^ **(E)**, and reducing power **(F)**. The data were expressed as means ± SD from three independent experiments.

Figure 3 represents the *in vitro* antioxidant activity of WSGP using DPPH free radical (Figure 3A), hydroxyl radical (Figure 3B), ABTS radical (Figure 3C) and superoxide anion scavenging abilities (Figure 3D), Fe^2+^ chelating capacity (Figure 3E), and reducing ability (Figure 3F). It can be seen that with the increased concentration of WSGP, the scavenging, chelating, and reducing abilities of WSGP solution in the range of 1–4 mg/mL were significantly improved. However, these abilities of WSGP were far weaker than that of Vc. In Figure 3A, when the WSGP concentration was raised from 0.125 to 2.0 mg/mL, its capacity to scavenge DPPH free radicals rose from 17.89 to 68.11%. With a concentration of 1.0 mg/mL, Vc was 88.91% effective at scavenging the DPPH free radical.

Hydroxyl radical is a reactive oxygen species. It can react with kinds of biomacromolecules (30). The oxidative damage of sugar molecules by hydroxyl radical can determine if the polysaccharide can scavenge hydroxyl radical. Figure 3B shows that the hydroxyl radical scavenging rate of WSGP ranged from 11.58% at a concentration of 0.125 mg/mL to 70.26% at a concentration of 4.0 mg/mL. Meanwhile, the hydroxyl radical scavenging capacity of 1 mg/mL Vc reached a peak of 88.75%.

A persistent blue-green solution with UV absorbance at 734 nm is produced when ABTS is oxidized by K2S2O8, forming ABTS radicals (31). When the ABTS radicals are neutralized by an antioxidant by accepting an electron or hydrogen, the absorbance drops in a proportionate fashion. Therefore, the antioxidant capacity can be determined based on the ABTS radical scavenging ability (Figure 3C). 0.125 mg/mL WSGP scavenged 18.74% free radical, while the Vc's clearance rate of ABTS radical at this concentration was as high as 73.65%. In addition, the increased concentration of Vc did not show a significant scavenging rate of ABTS radical, and 0.5 mg/mL Vc presented the strongest scavenging capacity, reaching 95.13%. Despite the weaker capacity of WSGP, 4.0 mg/mL WSGP showed a 92.03% of scavenging rate, which was closed to that of Vc.

As a kind of reactive oxygen species, superoxide anion can bind with NBT to generate a blue solution (32). The stronger scavenging ability results in less superoxide anion binding with NBT, thus leading to a lower absorbance value measured at 560 nm. Figure 3D demonstrates that the superoxide anion free radical clearance rate of 0.125 mg/mL WSGP was 28.83%, while Vc at this concentration was 85.79%. In addition, the scavenging rate of 4.0 mg/mL WSGP on superoxide anion free radical was only 68.52%, far lower than that of Vc. Garlic polysaccharide and its phosphorylated derivative were shown to have potent scavenging effects on hydroxyl radicals and superoxide anions in a prior investigation (5).

In the presence of ferrozine solution, the highly reactive ion Fe^2+^ forms a purple complex with peak absorbance at 562 nm. Absorbance drops correspondingly when Fe^2+^ is chelated with other ions (33). Figure 3E depicts the Fe^2+^ chelating capacity, 0.125 mg/mL WSGP on Fe^2+^ was only 8.36%. The chelating effectiveness of WSGP was 52.10% at a concentration of 4.0 mg/mL, whereas the chelating efficiency of Vc to Fe^2+^ reached 87.21% at a concentration of 0.5 mg/mL. The reducing ability of Vc increased linearly with its increased concentration. At 0.125 mg/mL, the reducing ability of WSGP and Vc was 0.15 and 0.38, respectively. The reducing ability of 4.0 mg/mL Vc reached 2.78. However, the reducing ability of 4.0 mg/mL WSGP was only 1.31 (Figure 3F).

Therefore, according to the effect of WSGP on the superoxide anion, hydroxyl, ABTS, and DPPH free radicals, reducing power, and Fe^2+^ chelating ability, it can be concluded that WSGP exhibited a certain *in vitro* antioxidant activity.

### 3.3. The effects of WSGP on LPS-induced RAW264.7 macrophages

#### 3.3.1. The effects of WSGP on cell viability

**Figure 4:**
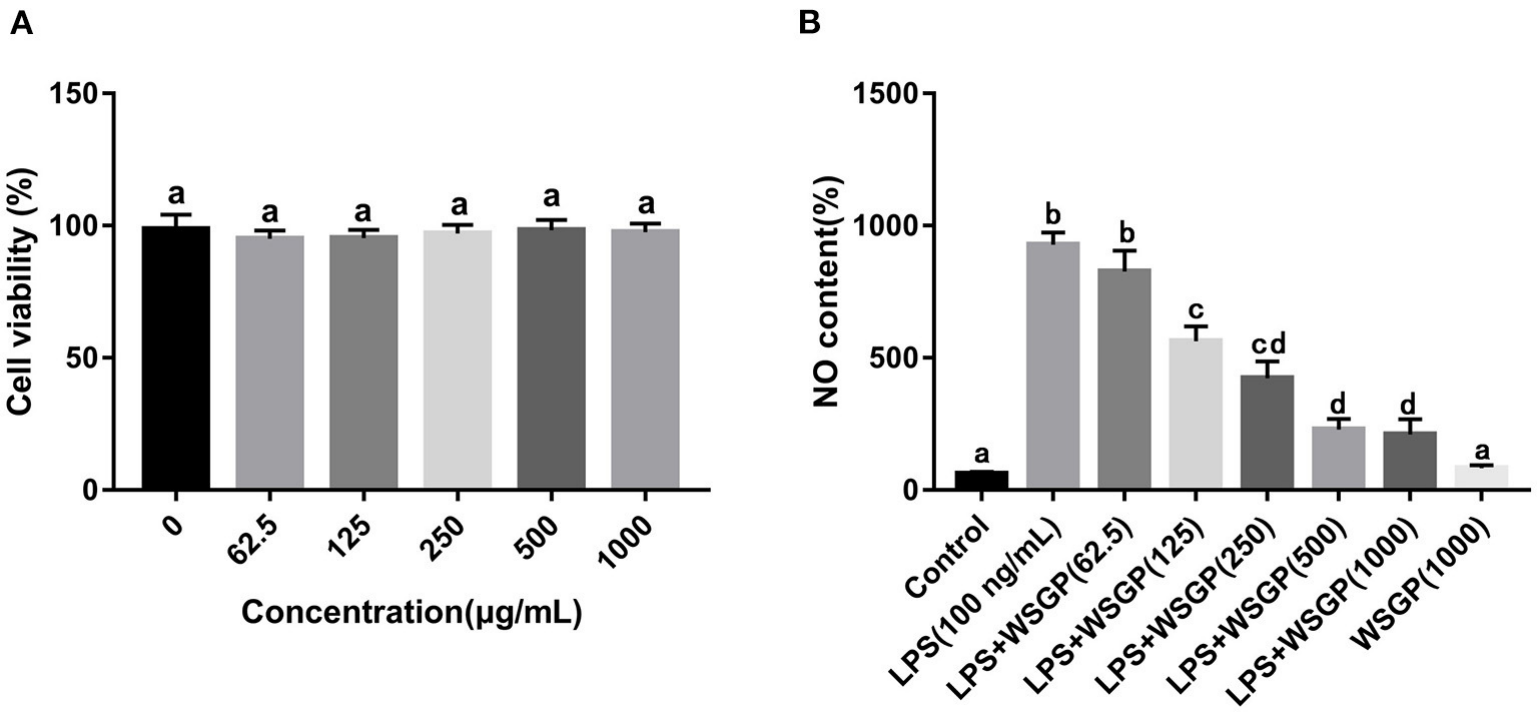
Effects of WSGP on cytotoxic **(A)** and LPS-induced NO production **(B)** in RAW264.7 macrophage cells. The data were expressed as means ± SD from three independent experiments. Values with different letters indicate significant difference (*p* < 0.05).

In order to screen the optimal concentration of WSGP on RAW264.7 macrophages, the MTT assay was employed to measure cell proliferation. As shown in Figure 4A, the cell survival rate of 62.5 μg/mL of WSGP-treated RAW264.7 was the lowest (95.18%), indicating that WSGP concentration had no toxic effect on RAW264.7 ranging between 0 and 1 mg/mL.

#### 3.3.2. The effect of WSGP on NO production

Gram-negative bacteria rely heavily on LPS as a component of their outer membrane. The recognition and signal transduction of LPS is very important in the autoimmune defense response of the human body (34). There is compelling evidence that RAW264.7 macrophages rely on the NF-κB and STAT3 signaling pathways to produce and secrete inflammatory mediators (NO, TNF-α, IL-1β, and IL-6) (35, 36). The amount of NO secretion is one of the important indicators to measure the severity of inflammatory response. Figure 4B demonstrates that NO generation in the model group driven by LPS alone was about 9 times greater than that in the control group (*p* < 0.05). The NO secretion in LPS-induced RAW264.7 macrophages was dose-dependently inhibited by 125–1,000 μg/mL of WSGP (*p* < 0.05). These findings showed that WSGP inhibited NO secretion in LPS-induced macrophages in a dose-dependent manner.

#### 3.3.3. The effect of WSGP on the secretion of inflammatory cytokines

**Figure 5:**
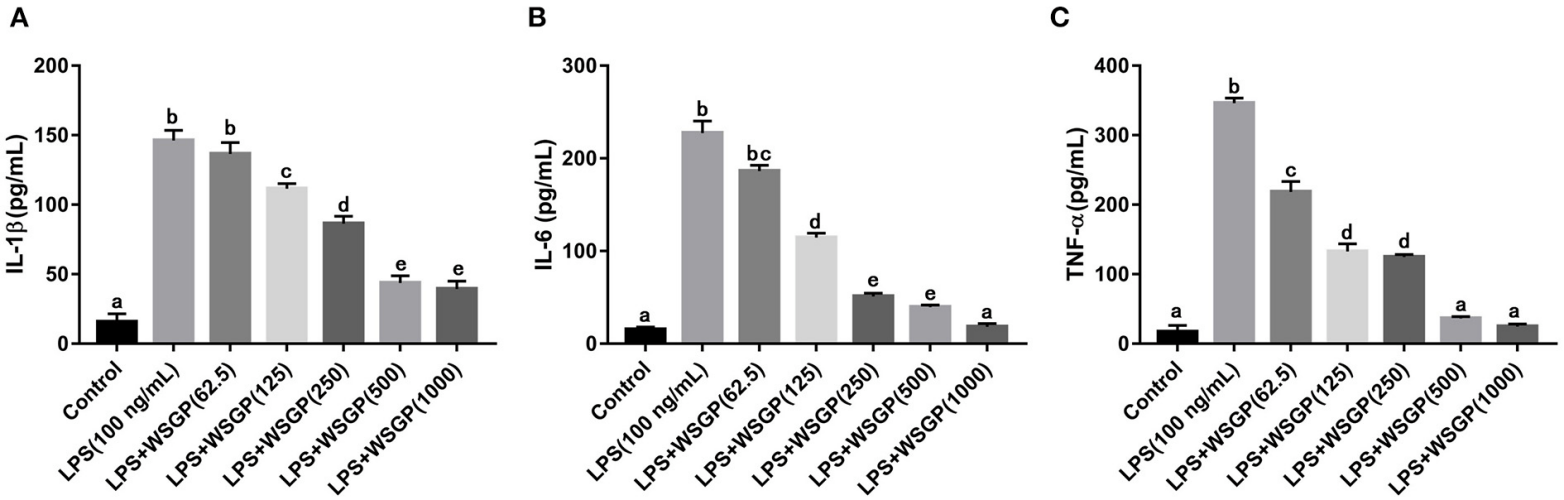
Effects of WSGP on proteins levels of IL-1β **(A)**, IL-6 **(B)**, and TNF-α **(C)** in LPS-induced RAW264.7 macrophages. The data were expressed as means ± SD from three independent experiments. Values with different letters indicate significant difference (*p* < 0.05).

The macrophage-secreted inflammatory cytokines are essential for fighting off infections and repairing tissue damage (37). The immune system relies on cytokines, which are a class of soluble polypeptide mediators secreted by a wide variety of cell types (38). Produced to mediate or increase the effects of proinflammatory cytokines on peripheral organs, such as IL-6 and IL-1β (39). During the acute inflammatory response, TNF-α overproduction is crucial to induce inflammatory genes, recruit and activate host immune cells (40). Herein, the releases of interleukins, particularly IL-6, TNF-α, and IL-1β induced by LPS were assessed (Figure 5). Findings showed that LPS induced cytokines secretion from RAW 264.7 macrophages. By contrast, LPS-induced production of IL-6, TNF-α, and IL-1β in RAW264.7 macrophages was substantially inhibited by 125–1,000 μg/mL WSGP intervention in a dose-dependent manner (*p* < 0.05).

**Figure 6:**
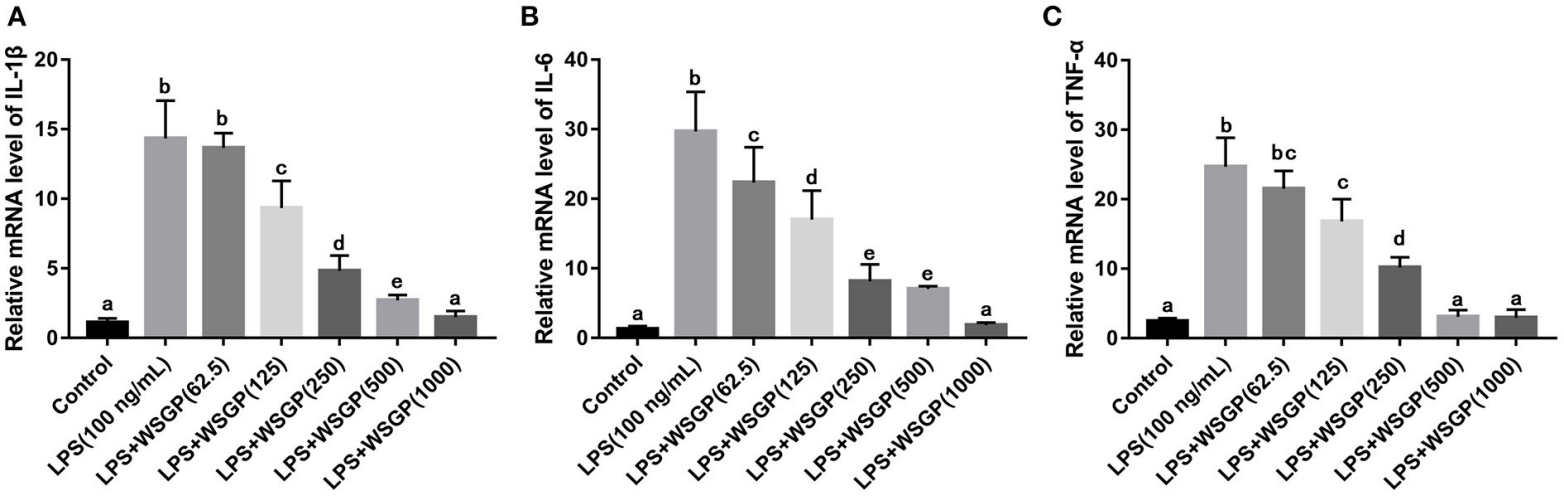
Effects of WSGP on mRNA levels of IL-1β **(A)**, IL-6 **(B)**, and TNF-α **(C)** in LPS-induced RAW264.7 macrophages. The data were expressed as means ± SD from three independent experiments. Values with different letters indicate significant difference (*p* < 0.05).

In addition, RT-PCR assays were performed to assess IL-1β, IL-6, and TNF-α mRNA expression levels. According to Figure 6, IL-1β, IL-6, and TNF-α mRNA levels were considerably enhanced in the model group administered with LPS alone compared with the control group (p < 0.01). The mRNA expression levels of IL-6, TNF-α, and IL-1β in LPS-induced RAW264.7 macrophages were considerably reduced after treatment with 125–1,000 μg/mL WSGP. This result was corresponding to the results measured by ELISA. This data added more support to the hypothesis that WSGP inhibits LPS-induced inflammatory cytokine release in RAW264.7 macrophages.

#### 3.3.4. The regulatory effect of WSGP on NF-κB/STAT3 signaling pathway

**Figure 7:**
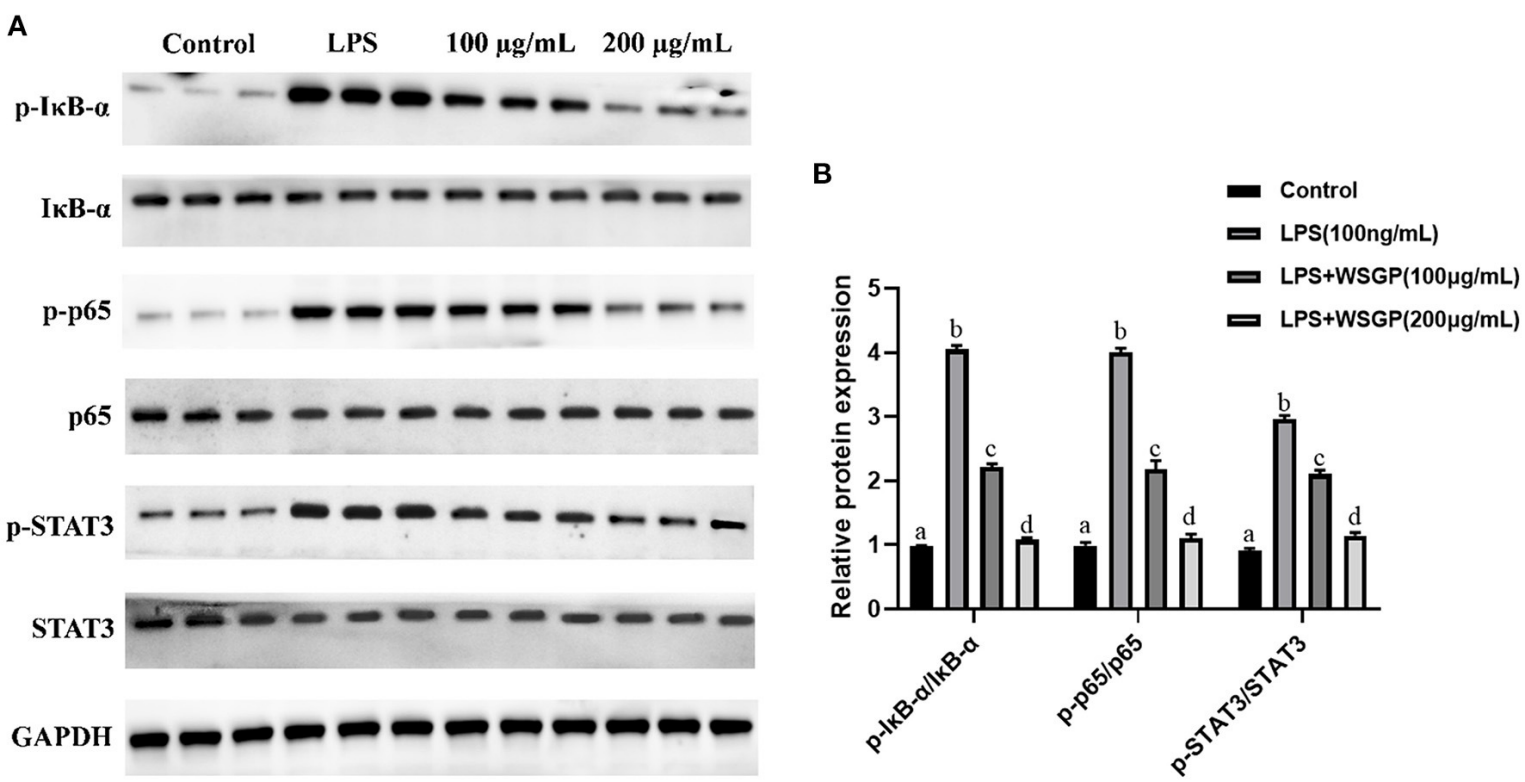
Inhibitory effect of WSGP on activation of NF-κB and STAT3 signaling pathways in LPS-induced RAW264.7 macrophages **(A)**. The data were expressed as means ± SD from three independent experiments **(B)**. The 100 and 200 μg/mL groups respectively represented different doses of WSGP intervention while induced by LPS.

Activation of inflammatory signaling pathways including NF-κB and STAT3 has been demonstrated to enhance the release of inflammatory cytokines, leading to an escalation of inflammation (41–43). The NF-κB is essential for the promotion of the acute inflammatory process and the regulation of the expression of genes involved in inflammatory responses, such as chemokines and cytokines (44). IκB-α is a critical regulator of the transcription factor NF-κB that could activate a wide range of genes involved in immunological and inflammatory responses. Down-regulating IκB-α levels and localization can lead to several diseases, such as chronic inflammatory disorders and cancers (45). STAT3, a well-characterized protein with 92 kDa, has been shown to be activated by epidermal growth factor and IL-6 (46). Herein, Figure 7A shows the inhibitory effect of WSGP on phosphorylation activation of target proteins in the NF-κB and STAT3 signaling pathways. Figure 7B shows that compared to the control group, LPS substantially enhanced the protein levels of p-IκB-α, p-P65, and p-STAT3 (*p* < 0.01), suggesting that LPS could activate the NF-κB and STAT3 signaling pathways by promoting the phosphorylation of IκB-α, P65, and STAT3, and consequently leading to the release of inflammatory cytokines such as IL-6 and TNF-α. However, LPS-induced RAW264.7 macrophages treated with 100 or 200 ng/mL WSGP showed a dose-dependent suppression of p-IκB-α, p-P65, and p-STAT3 protein expressions (*p* < 0.01). Moreover, the inhibitory effect of 200 μg/mL WSGP on p-IκB-α, p-P65, and p-STAT3 protein expression was better than that of 100 μg/mL WSGP (*p* < 0.05). These results further supported that LPS significantly increased protein and mRNA expression levels of IL-6, TNF-α, and IL-1β, while WSGP intervention could reverse this activation (*p* < 0.05). Taken together, these findings indicate that WSGP may have an anti-inflammatory effect by blocking the activation of the NF-κB and STAT3 signaling pathway in LPS-induced RAW264.7 macrophages, consequently decreasing the secretion and gene expression of inflammatory cytokines. This research uses Graphical abstract to briefly outline the chemical molecular mechanism through which WSGP exerts its anti-inflammatory activity.

## 4. Conclusions

The small molecular garlic polysaccharide WSGP used in this experiment exhibited antioxidant activity and has significant DPPH, hydroxyl, superoxide anion, ABTS free radical scavenging powers, Fe2+ chelating ability and reducing power. Meanwhile, the generation of NO and the mRNA and protein expression levels of IL-1β, IL-6, and TNF-α were all dramatically decreased in LPS-stimulated RAW264.7 macrophages after treatment with WSGP. In addition, the mechanisms study further elucidated that WSGP might inhibit the activation of NF-κB and STAT3 signaling pathways by discouraging the phosphorylation of IκB-α, P65, and STAT3, thereby inhibiting the transcription of downstream pro-inflammatory factors. This study gives scientific evidence that small molecular garlic polysaccharides have significant antioxidant and anti-inflammatory activities, which is going to be extremely important in the study of functional foods and nutritional supplements. However, further mechanism studies *in vivo* are required to validate its anti-inflammatory properties.

## Data availability statement

The original contributions presented in the study are included in the article/supplementary material, further inquiries can be directed to the corresponding authors.

## Author contributions

CC and XS: conceptualization. XS and JL: methodology and software. XS and XZ: validation. XS and XW: formal analysis. XS and HZ: investigation. CC and XW: resources and project administration. XS: data curation and writing—original draft preparation. JL, YW, and CS: writing—review and editing. XS and XO: visualization. CC: supervision and funding acquisition. All authors have read and agreed to the published version of the manuscript.
